# Integrated bioinformatics analysis of validated and circulating miRNAs in ovarian cancer

**DOI:** 10.5808/gi.21067

**Published:** 2022-06-30

**Authors:** Berkcan Dogan, Ece Gumusoglu, Ege Ulgen, Osman Ugur Sezerman, Tuba Gunel

**Affiliations:** 1 Department of Medical Genetics, Faculty of Medicine, Bursa Uludag University, Bursa 16059, Turkey; 2 Department of Translational Medicine, Institute of Health Sciences, Bursa Uludag University, Bursa 16059, Turkey; 3 Department of Molecular Biology and Genetics, Faculty of Science, Istanbul University, Istanbul 34134, Turkey; 4 Department of Biostatistics and Medical Informatics, School of Medicine, Acibadem Mehmet Ali Aydinlar University, Istanbul 34750, Turkey

**Keywords:** integrative analysis, KEGG pathway, microRNAs, ovarian cancer, pathfindR

## Abstract

Recent studies have focused on the early detection of ovarian cancer (OC) using tumor materials by liquid biopsy. The mechanisms of microRNAs (miRNAs) to impact OC and signaling pathways are still unknown. This study aims to reliably perform functional analysis of previously validated circulating miRNAs' target genes by using pathfindR. Also, overall survival and pathological stage analyses were evaluated with miRNAs' target genes which are common in the The Cancer Genome Atlas and GTEx datasets. Our previous studies have validated three downregulated miRNAs (hsa-miR-885-5p, hsa-miR-1909-5p, and hsa-let7d-3p) having a diagnostic value in OC patients' sera, with high-throughput techniques. The predicted target genes of these miRNAs were retrieved from the miRDB database (v6.0). Active-subnetwork-oriented Kyoto Encyclopedia of Genes and Genomes (KEGG) pathway enrichment analysis was conducted by pathfindR using the target genes. Enrichment of KEGG pathways assessed by the analysis of pathfindR indicated that 24 pathways were related to the target genes. Ubiquitin-mediated proteolysis, spliceosome and Notch signaling pathway were the top three pathways with the lowest p-values (p < 0.001). Ninety-three common genes were found to be differentially expressed (p < 0.05) in the datasets. No significant genes were found to be significant in the analysis of overall survival analyses, but 24 genes were found to be significant with pathological stages analysis (p < 0.05). The findings of our study provide *in-silico* evidence that validated circulating miRNAs' target genes and enriched pathways are related to OC and have potential roles in theranostics applications. Further experimental investigations are required to validate our results which will ultimately provide a new perspective for translational applications in OC management.

## Introduction

Despite the numerous research and clinical studies, ovarian cancer (OC) still has a high mortality rate among gynecological cancers since lack of effective biomarkers for early detection and prognosis [1-3]. Also, the treatment efficiency of OC is low depending on multiple challenges such as late diagnosis, lack of reliable markers, the development of resistance to current therapeutics, and phenotype of heterogeneity [1,4]. For these challenges of OC, new studies need to reveal underlying molecular mechanisms, and to discover molecular biomarkers for early diagnosis, prevention, and targeted therapy [4].

MicroRNAs (miRNAs) are small non-coding RNAs that are about ~22 nucleotides in length. Their function is transcriptional and post-transcriptional regulation of gene expression by targeting mRNAs [5,6]. According to the effect on cancer, there are two types of miRNAs which are tumor suppressor miRNAs and oncomiRs. Depending on the type of cancer, oncomiRs or tumor suppressor miRNAs are inhibited or stimulated, respectively [7]. Especially, dysregulations of specific miRNAs affect cancer cell proliferation, differentiation, metastasis, and recurrence formation [8,9]. Various miRNAs have been shown to play different roles in OC. The mechanisms of miRNAs to impact OC and signaling pathways are still unknown. [2,10].

Many experimental studies have verified the detected interactions with bioinformatics analysis and proved the accuracy and predictivity with *in-silico* tools or databases. Although significant progress has been made about *in-silico* analysis in evaluating miRNAs, the need for new tools/databases is increasing day by day. It is also because of the limitations in existing tools/databases, that has increased with the development of high-throughput miRNAs technologies to analyze miRNA [11,12].

In this study, pathfindR was used for enrichment analysis of target genes. PathfindR uses active subnetworks, where an active subnetwork can be defined as a subnetwork of interconnected genes in a protein-protein interaction network (PIN), predominantly consisting of significantly altered genes. The tool initially maps the input genes with significance values onto the PIN and identifies active subnetworks, then it performs enrichment analysis on the identified subnetwork gene sets. In general, enrichment approaches overlook the relational information captured in the PIN and the genes neighboring the significant genes are not considered. By identifying active subnetworks, pathfindR exploits interaction information to enhance enrichment analysis. Active subnetworks allow the inclusion of possibly relevant genes that are not significant but connect significant genes in the PIN, and, in turn, the identification of phenotype-associated connected significant subnetworks [13]. This aids pathfindR uncover relevant mechanisms underlying the studied disease/phenotype.

**Fig. 1.**
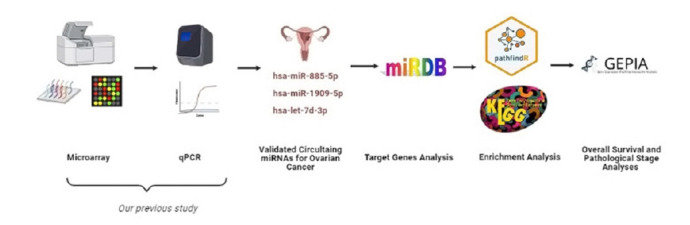
The workflow for bioinformatics analysis of miRNAs. GEPIA, Gene Expression Profiling Interactive Analysis; qPCR, quantitative polymerase chain reaction.

In this study, we developed an *in-silico* approach to evaluate target genes of OC-related circulating and previously validated miRNAs which may be targeted for therapeutic approaches and utilized for OC management and diagnosis (Fig. 1).

## Methods

### Identification of the targets genes of validated miRNAs

In our functional analyses, we used hsa-miR-885-5p, hsa-miR-1909-5p, and hsa-let7d-3p which were previously defined by our group as the dysregulated miRNAs that can be a candidate biomarker for OC. These candidate miRNAs, which were determined by microarray, were validated by quantitative polymerase chain reaction (qPCR). Both microarray and qPCR results showed that these three miRNAs were downregulated in the OC group compared with healthy individuals [14,15].

Target genes of validated circulating miRNAs were examined by using the miRDB online database (http://www.mirdb.org). As one of the miRNA-target predictions and the functional annotations databases, miRDB provides access to miRNA-target genes and functions of five different species: human, mouse, rat, dog, chicken. All targets in the database were acquired from the MirTarget database, which was a bioinformatics tool developed with analyzing thousands of miRNA-target interactions obtained from high-throughput techniques. By integrating target prediction and gene ontology enrichment analyses, miRDB presents a streamlined pipeline for quickly identifying miRNA functions [16].

### Functional and pathway enrichment analyses

Using all (i.e., union of) target genes of the validated miRNAs, active-subnetwork-oriented Kyoto Encyclopedia of Genes and Genomes (KEGG) pathway enrichment analysis was performed using pathfindR. The tool pathfindR identifies gene sets that form active subnetworks in a PIN using a list of genes. Afterwards, it performs pathway enrichment analysis. An active subnetwork can be defined as a group of interconnected genes in a PIN that predominantly consists of significantly altered genes. Active subnetworks define distinct disease-associated sets of interacting genes. By incorporating interaction information, pathfindR yields more relevant enrichment results.

For assigning a significance value for each target gene (for use with pathfindR), initially, all *Homo sapiens* miRNA-target gene scores were obtained from miRDB (v6.0). The significance for each target gene was defined as the probability of observing a score greater than or equal to the score of this target gene over all *H. sapiens* miRNA-target gene scores [i.e., P(x ≥ observed score)]. For genes that are targeted by more than one miRNA, the lowest significance was kept. The final list of target genes-significance values was, then, filtered keeping genes with significance ≤ 0.5 (corresponds to a score of 67). The significance value used for pathfindR indicates what proportion of all scores (across all *H. sapiens* miRNA-target gene scores) was as high or higher than the observed score for a given miRNA-target gene pair. The threshold proxy significance value of 0.5 was an ad-hoc choice corresponding to a score of 67, which was roughly equivalent to the threshold value suggested by miRDB for obtaining high-confidence miRNA-target gene pairs.

### Determination of differentially expressed target genes

The expressions of the target genes were enriched in The Cancer Genome Atlas and Genotype-Tissue Expression (GTEx) datasets by using GEPIA (Gene Expression Profiling Interactive Analysis) (http://gepia.cancer-pku.cn). GEPIA provides customizable functions, such as tumor and/or normal differential expression analysis, profiling according to cancer types or pathological stages, patient survival analysis, detection of gene expression similarities, correlation analysis and dimensionality reduction analysis using RNA sequencing (RNA-seq) data of The Cancer Genome Atlas (TCGA) and GTEx projects [17].

All target genes enriched were analyzed separately by using the Expression DIY feature and performed for the RNA-seq data of TCGA and GTEx datasets by using the ANOVA test. For each gene expression features of box plots were set as |Log2FC| cutoff: 1 and p-value cutoff: 0,01, jitter size: 0,4 and log2 (TPM + 1) for log-scale. Genes with a p-value <0.05 were identified as differentially expressed target genes.

### Overall survival and pathological stage analyses

The associations between expression signatures of shared genes and overall survival and pathological stage analyses were performed in the TCGA and GTEx dataset by the GEPIA platform. For overall survival plot analysis used log-rank test, also known as the Mantel-Cox test, for the hypothesis test and cohorts' thresholds adjusted. The Cox proportional hazard ratio and the 95% confidence interval information are included in the survival plot. The method for differentially expressed target gene analysis is one-way ANOVA, using the pathological stage as variable for calculating differential expression. The expression data are first log2(TPM+1) transformed for differential analysis. They included further statistical analysis, Benjamini and Hochberg's false discovery rate (FDR) adjusted p-value (q-value) < 0.05 was identified as statistically significant. Genes with FDR p-value (q-value) < 0.05 were identified as significant association with overall survival or pathological stage.

## Results

### Target genes analysis of miRNAs

**Fig. 2.**
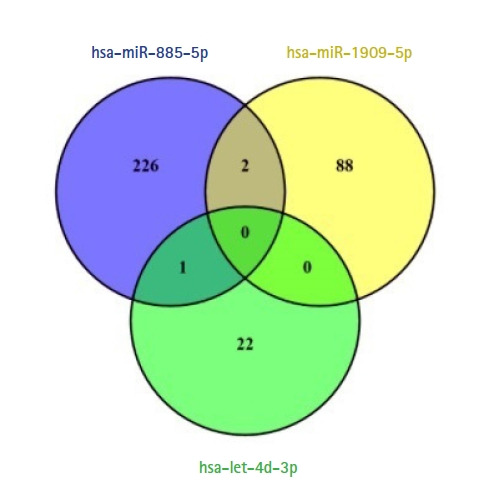
Venn diagram of common and overlapping target genes identified for the three miRNAs (score ≥ 67).

Target genes of validated circulating miRNAs were comprehensively analyzed by miRDB database. Target genes were retrieved separately determined for each miRNA. No specific filters were applied for target gene prediction in miRDB database. Respectively, 422, 230, and 44 target genes were identified with mining target genes of hsa-miR-885-5p, hsa-miR-1909-5p, and hsa-let-7d-3p. Target genes were filtered by a score of 67 for significance values. After filtering, the number of target genes detected for hsa-miR-885-5p, hsa-miR-1909-5p, and hsa-let-7d-3p were respectively 229, 90, and 23 (Table 1). While two common target genes were found in hsa-miR-885-5p and hsa-miR-1909-5p: *ERICH3* (glutamate rich 3) and *CAPRIN1* (cell cycle associated protein 1), one common target gene were observed in hsa-miR-885-5p and hsa-let-7d-3p: SEC24D (SEC24 homolog D, COPII coat complex component). No common target gene was found for all three miRNAs. The numbers of target and common genes are shown in Fig. 2. Venn diagrams were drawn by using VENNY 2.1 (https://bioinfogp.cnb.csic.es/tools/venny) online web tool.

### Pathway enrichment analyses of target genes of miRNAs

**Fig. 3.**
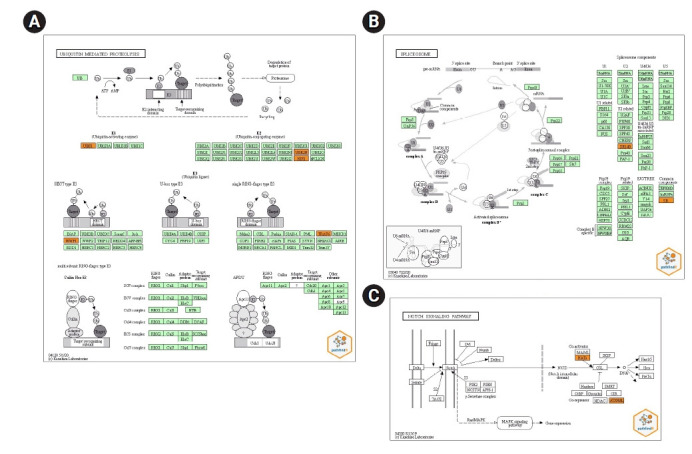
Kyoto Encyclopedia of Genes and Genomes (KEGG) pathway diagrams of ubiquitin-mediated proteolysis (A), spliceosome (B), and Notch signaling pathway (C). Genes targeted by at least one validated miRNA are indicated in orange color.

Performing active-subnetwork-oriented enrichment analysis via pathfindR using the union of miRNA-target genes (339 genes in total), 24 enriched KEGG pathways were identified (Table 2). The top three pathways were ubiquitin-mediated proteolysis, spliceosome, and Notch signaling pathway according to p-values. The pathway diagrams for these pathways, in which the target genes of our dysregulated miRNAs are indicated by orange color, are presented in Fig. 3. It is found that a total of ten different target genes were involved in these pathways. A part of the target genes which are *TRAF6* (TNF receptor associated factor 6), *UBE2N* (ubiquitin conjugating enzyme E2 N), *UBA7* (ubiquitin like modifier activating enzyme 7), *UBE2K* (ubiquitin conjugating enzyme E2 K), *WWP1* (WW domain containing E3 ubiquitin protein ligase 1) are ubiquitin-mediated proteolysis (Fig. 3A) whereas, *SRSF2* (serine and arginine rich splicing factor 2), *SRSF6* (serine and arginine rich splicing factor 6), and *U2SURP* (U2 SnRNP associated SURP domain containing) are involved in spliceosome pathway (Fig. 3B). There are only two genes in the Notch pathway: *ATXN1L* (ataxin 1 like) and *KAT2B* (lysine acetyltransferase 2B) (Fig. 3C). While nine of these ten genes are (*UBE2N*, *UBE2K*, *WWP1*, *TRAF6*, *U2SURP*, *SRSF2*, *SRSF6* *KAT2B*, and *ATXN1L*) targeted by hsa-miR-885-5p, only the *UBA7* gene is targeted by hsa-miR-1909-5p. There are no target genes of hsa-let-7d-3p associated within three pathways.

### Analysis of differentially expressed target genes

Differentially expressed target gene analysis was conducted to examine the expressions of identified target genes of miRNAs in the TCGA and GTEx datasets. In union of miRNA-target genes (339 genes in total), 93 genes were found to be differentially expressed (p < 0.05) in TCGA and GTEx datasets of when compared to cancer tissues with paired normal tissues (Table 3). None of the common genes (*ERICH, CAPRIN1*, and *SEC24D*) among target genes were significant in TCGA and GTEx datasets. While miR-885-5p targets 64 out of 93 genes, miR-1909-5p regulates 20 and let-7d-3p 9 target genes.

### Overall survival and pathological stage analyses

**Fig. 4.**
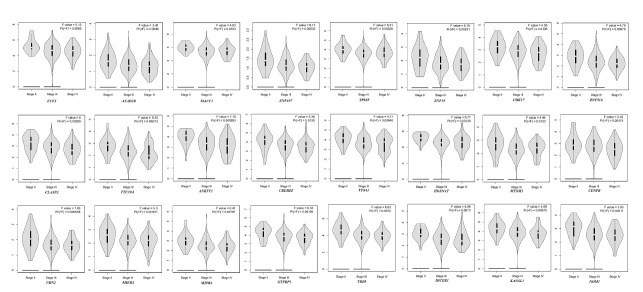
Pathological stage analysis of differently expressed target genes in the ovarian cancer dataset using the GEPIA (Gene Expression Profiling Interactive Analysis) platform (p < 0.05). Pr(>F) values were adjusted to Benjamini and Hochberg's false discovery rate test.

The associations with overall survival outcomes and pathological stage analysis for expression signatures of 93 genes in the TCGA and GTEx datasets were performed by the GEPIA (Table 3). After FDR tests, no significant genes were found to be significant in the analysis of overall survival analyses, but 24 genes were found to be significant with pathological stages analyses (p < 0.05). No significant differentially expressed target genes were found to be with overall survival after statistical analyses. However, 24 differentially expressed target genes were significantly associated pathological stage of OC (Fig. 4). Of 24 genes, *ZNF407* (zinc finger protein 407) and *UBN2* (ubinuclein 2) genes had the lowest p-value for their relationship with pathological stages for the TCGA and GTEx datasets (p = 0.017856) (Table 3).

## Discusssion

Recently, a considerable number of research have been made to determine the effect of miRNAs regulate cancer hallmarks and to develop the early diagnosis and prognosis of cancer [18]. Nevertheless, several challenges such as various sampling methods, sample size, detection techniques, gender, and ethnicity or genetic background, affect the reliance on utilizing miRNAs as biomarkers [1,3,10,19]. One of the fundamental research areas for easily detectable, non-invasive, sensitive, and specific miRNA-based biomarker discoveries are of a great value for accurate and effective early diagnosis, risk prediction, prognosis, recurrence, and effective management of OC [8,10,12].

Identification of miRNAs' target genes has been the focus of computational biology for the last few years. Since detecting all possible miRNA targets with high-throughput technologies is laborious and costly, a wide variety of computational resources has been developed. The methodologies used by databases range from evolutionary conservation evaluations of putative miRNA binding sites to machine learning and classification algorithms. Continuous improvement is needed to develop new tools/databases to accurate predictions of miRNA targets [20,21]. One of these databases is miRDB online database and determines miRNA-target prediction and functional annotations. Estimating that 3.5 million target genes were regulated by 7,000 miRNAs in human, mouse, rat, dog, and chicken, in the latest version of miRDB (v6.0) major updated in 2019. In miRDB, miRNA binding and target down-regulation features used to predict miRNA targets with machine learning methods, generates a prediction scores are in the range of 0–100, and candidate genes with scores ≥ 50 are presented as predicted miRNA targets [16].

The pathfindR tool was used for the enrichment analysis of target genes within the scope of the study. While pathfindR allows better identification of disease-related pathways, it should be noted that the tool requires a significance level per each input gene. To overcome this limitation, we followed an approach where we used the scores for miRNA-target gene pairs to calculate a significance level. Additionally, some genes (usually a small proportion) that are not in the PIN (that do not have any curated interactions) have to be discarded, resulting in the loss of possibly relevant genes.

In our previous studies, three downregulated circulating miRNAs (hsa-miR-1909-5p, hsa-miR-885-5p, and hsa-let-7d-3p) in OC patients were consistently validated by comparison with healthy individuals. In these studies, circulating miRNAs, which were determined to be dysregulated by microarray, were then validated with the qPCR [14,15]. In this study, we predicted the validated circulating miRNAs' target genes with miRDB database and performed functional analysis by pathfindR tool to understand the pathogenesis of OC especially the molecular mechanisms of its development. Also, overall survival and pathological stage analyses were evaluated with differentially miRNAs' target genes which are commonly found in the TCGA and GTEx datasets.

Two hundred and twenty-nine, 90, and 23 of target genes of hsa-miR-1909-5p, hsa-miR-885-5p, and hsa-let-7d-3p were separately determined via miRDB (score ≥ 67), respectively (Fig. 2). Enriched KEGG pathways of target genes were performed with pathfindR tool. Totally, 339 genes included in active-subnetwork-oriented enrichment analysis and as a result, 24 enriched KEGG pathways were identified (Table 2). Top three pathways with the lowest p-values are ubiquitin-mediated proteolysis (p < 0.001), spliceosome (p < 0.001), and Notch signaling pathway (p < 0.001), respectively (Fig. 3). *TRAF6*, *UBA7*, *UBE2K*, *UBE2N*, and *WWP1* genes regulate the ubiquitin-mediated proteolysis pathway. While *SRSF2*, *SRSF6*, and *U2SURP* are involved in the spliceosome, *ATXN1L* and *KAT2B* are adjusted to the Notch pathway. Although hsa-miR-885-5p targets *ATXN1L*, *KAT2B*, *SRSF2*, *SRSF6*, *TRAF6*, *UBE2K*, *UBE2N*, *U2SURP*, and *WWP1* genes, hsa-miR-1909-5p just targets *UBA7* gene. No target genes of hsa-let-7d-3p were found to be associated with these three pathways.

Our pathway analysis determined different pathways that can be related to the OC development. Of these pathways, only ubiquitin-mediated proteolysis, spliceosome, and Notch signaling pathway showed that their p-value is less than 0.001. Besides the effects of these pathways on OC, they also have roles on other cancer types. Firstly, ubiquitin-mediated proteolysis is an essential mechanism that is responsible for 80%–90% of intracellular protein degradation and is involved in many cellular processes, including tumorigenesis, tumor survival and apoptosis [22,23]. Ubiquitin-mediated pathway modulates *BRCA1/2*, p53, *ERBB2* gene expressions, ERK pathway, cyclin-dependent cell cycle regulation which are related to OC [24]. Bazzaro et al. [25] claimed that upregulation of proteasome subunit levels occurs in OC and proteasome inhibitors may have utility in the treatment of OC. Secondly, splicing mechanism is a crucial process that regulates cellular proliferation, differentiation, and survival [26]. Dysregulation of splicing processes and splicing factor genes contribute to cancer, including OC [27,28]. Especially, splicing machinery mutations especially contribute to tumorigenesis. Additionally, it is critical to understand that the molecular mechanism of RNA splicing is causing the development of drug resistance in cancer treatment [26]. Regulation of the relationship between splicing and cancer can be led to splicing-based therapies for cancer treatment [29]. Thirdly, Notch signaling pathway regulates not only cell self renewal and differentiation but also a cell to cell communication [30]. Upregulation of Notch signaling pathway proteins have been identified in OC [31]. Several studies have revealed that it is related to poor overall and disease free survival time, and more advanced stages [30,32]. The Notch signaling pathway plays a specific role in deregulation of signaling cascade has been associated with OC. Targeted therapy against Notch pathway activation can offer clinical benefit to OC [30,31].

According to these studies, our findings also indicate that our dysregulated miRNAs are significant in OC via pathway related-target genes. Briefly, circulating downregulated miRNAs could not suppress their target genes in these pathways, hence, have activated these pathways, resulting in the development of OC. As we can see from the previous studies, these pathways were found to be related with OC. We have discovered target genes of three downregulated circulating miRNAs (hsa-miR-1909-5p, hsa-miR-885-5p, and hsa-let-7d-3p) and also related-pathways of these genes. We propose novel mechanisms between miRNAs, target genes and OC that have not been elucidated previously using pathfindR. Our findings can be used as a diagnostic tool in OC. Our perspective on *ATXN1L*, *KAT2B*, *SRSF2*, *SRSF6*, *TRAF6*, *UBA7*, *UBE2N*, *UBE2K*, *U2SURP*, *WWP1* and in OC will promote more extensive research on the molecular mechanisms of hsa-miR-1909-5p, hsa-miR-885-5p, and hsa-let-7d-3p and provide a reference for improving the clinical outcome of OC.

The development of prognostic multigene classification protocols can benefit the understanding of tumor biology as well as the prediction of cancer progression and treatment strategies. One critical issue is determining the properly combining the genes [33]. However, studies on the overall survival-related profiles in OC patients have progressed, whereas there have been no large-scale studies based on multicenter validation of gene expression profiles for prediction of disease progression or recurrence in OC patients [34]. Furthermore, pathological staging, which can be determined after surgery and examination of the removed tumor tissue, is likely to be more accurate than clinical staging because it provides direct insight into the extent and nature of the disease [35]. Additionally, differentially expressed miRNAs' target genes, which were identified as multiple candidate genes in OC, were integrated our multigene analysis into the overall survival outcomes and pathological stage analysis using TCGA and GTEx datasets (Table 3). The hsa-miR-885-5p, which was the most gene-targeting score (n = 64) and the most associated miRNA with OC, followed by the hsa-miR-1909-5p and hsa-let-7d-3p miRNAs which were gene-targeting scores (n = 20) and (n = 9), respectively. After FDR analysis, no significant differentially expressed target genes were found to be associated with overall survival. However, we found 24 genes that were targeted by three miRNAs identified in the pathological stage of OC (Fig. 4). Notably, *ZNF407* and *UBN2* had the lowest p-values for their association with OC pathological stages (p = 0.017856) (Table 3). The molecular role and mechanism of *ZNF407* and *UBN2* in the development and progression of OC is not well understood. Missense mutations in *ZNF407* affect tumor progression in gastrointestinal stromal tumors were reported [36]. Moreover, Tan et al. [37] showed that *ZNF407* functiones as a promotive factor in colorectal cancer metastasis and accelerates cell proliferation by regulating phosphoinositide 3-kinase/AKT-mediate pathway. *UBN2* is widely expressed in tumor tissues and encodes a nuclear protein that interacts with viral and cellular transcription factors [38]. Zhao et al. [39] suggested that high *UBN2* protein expression is an independent prognostic marker to identify patients with poor clinical outcomes in colorectal cancer by affecting the Ras/MAPK pathway. Thus, these genes are likely to be facilitated in therapeutic approaches for OC.

In conclusion, we offer *in-silico* evidence that validated circulating miRNAs' target genes and enriched pathways are related to OC and have potential roles in theranostics applications. Especially, enrichment pathways and pathological stage-related genes can be combined with validated miRNAs, their multiple analysis can further enhance the molecular etiology of the OC and also can be employed in future research for biomarker and drug development related to OC. Further experimental investigations are required to validate our results which will ultimately provide a new perspective for translational applications in OC management. Our study will allow a greater understanding of broader clinical application prospects.

## Figures and Tables

**Table 1.** Target genes determined in the miRDB database (score ≥ 67, for representation purposes)

miRNAs	Target genes
hsa-miR-885-5p	*GALNT3, ZNF367, C9orf3, TMEM135, CTNNB1, ZNF354A, OXR1, ZFP91, LRP4, CPEB2, SYTL4, SLC11A2, ZNF281, PGK2, ASCL4, CAMK4, FAM81A, KIF21A, MXD1, LCP1, ATXN1L, C8orf88, CCDC71L, UPF2, ADD1, FAM241A, GPR68, MAN1C1, ELAVL1, ZADH2, PANK3, PFDN4, JAZF1, OR51E1, ECE1, FURIN, CDC73, FNTA, MAP4K3, UBTD2, LACTB, ATAD2B, ENKUR, TMEM41A, FAM98A, KAT2B, EMILIN2, CPSF6, NLRP1, KIAA0825, OSBPL6, ZFY, MAGT1, ZBTB34, NUDT21, CEP170, CPEB3, ING3, SNAPC3, ARID2, PDE10A, NUDCD2, MTMR9, ASB5, ZC3H12C, MAN1A2, PCSK5, WWP1, PARVA, DPY19L1, TDRD6, PDCD6IP, VSTM2A, PROK2, ZC3HAV1, SH2D1A, HSFY2, LAMC2, HSFY1, MACF1, WNT5A, VPS13A, GTF2H3, ZNF407, MEX3D, TMEFF2, PHF6, TSC22D2, SLC33A1, SRSF6, DNAJC15, STRIP2, EIF1AX, EIF1AY, SMIM13, TPMT, RFX7, CEBPD, ZNF10, PBOV1, NSUN5, SEMA6A, UBE2N, CXADR, CDK17, TMEM185B, TCTEX1D1, PDK1, CPSF7, SLC35D1, ZNF516, PARG, RAD54B, RET, NME5, SLC35F5, HMBOX1, SAMD12, FSBP, CCDC182, SMARCE1, CLASP2, LRRK2, LYPLA1, B3GLCT, TMEM41B, YWHAE, IMPACT, HSBP1, PPP3R1, HINT3, FSD1L, KIF1C, MRPL2, SH3BGRL2, TTC30A, ANKFY1, CDK14, ERICH3, ZFHX4, HMGB1, ZFX, TMPRSS11A, UBE2K, NR2C1, TMEM230, PRRG1, MRPS10, KLHL29, SRSF2, ETFBKMT, GOPC, HRASLS5, AIG1, SLC38A2, PAK5, NUP50, RBPMS, GPR75, DGKH, LPAR1, CALN1, ZNF80, ANKS4B, STMN2, NR5A2, BMP3, EYA1, CREBZF, SEC24D, TRIM7, LRRC40, TRAF6, U2SURP, EZR, IL6ST, VPS41, JAKMIP3, FREM2, CCR9, SYPL1, RPGRIP1L, RBBP4, CAPRIN1, MYT1, EYA3, POU4F2, RSPH3, CTNNA3, FZD10, HGSNAT, ARPP21, TNRC6C, REL, ERBIN, SEMA6D, KHDRBS2, RBM14, TGFBR1, AP2M1, MYSM1, WDR43, URI1, TBCC, TVP23B, USF3, GNB4, ABCB8, CENPK, USP14, CLVS2, CD59, TOX, PLEKHA5, C10orf25, OPA1, ABHD17C, SOAT1, UBN2, YWHAH, CCDC155, IL6R, FAM126A, DCUN1D5, MIER3, RHOQ, DSTN, HIPK3, MDM4*
hsa-miR-1909-5p	*F7, PTPN4, RCBTB2, MEF2C, TAF5, TSPAN16, CCDC113, COX11, SHOC2, KBTBD12, BCO2, KIF3B, TIGD2, POM121, ERICH3, BLOC1S5, MMD2, RAPGEFL1, YY1, B4GALT1, AEBP2, BAZ1A, PLCXD2, C2orf48, C4orf19, SERF2, GPX4, MOB4, ARID4A, ADGRL3, ZNF275, CREB5, PIGZ, TBC1D10A, FAM83G, ERBB3, EIF4E2, YES1, GLP2R, METTL7B, IL21R, RPGR, COL8A1, KCNK15, GGA2, COPS7B, ZKSCAN1, SNTB2, MFHAS1, CEMP1, GTPBP1, TRIO, CNGB3, GMPR2, ARPIN-AP3S2, MEOX2, ABHD2, AP3S2, APC, ZNF687, YAE1, STMND1, SCAMP5, DICER1, FCRL1, RAB36, SGCD, NOL12, FMC1, TGFA, CNKSR2, UBA7, ECHDC2, TTC16, PTPA, SLC35E4, BAP1, CDKN1B, PEX19, DNAJC11, EIF4EBP2, CHST9, KCTD16, MEP1A, GDF6, CAPRIN1, RP1L1, RIMKLA, ARL8B, STX5*
hsa-let-7d-3p	*MEX3C, SEC24D, BEND2, PTGIS, PARP11, HMGA2, SH3RF1, PRKACB, GSTK1, PHF14, EXOSC7, SIGLEC6, CYP4Z1, KANSL1, NOM1, ABCA8, ZNF486, CASD1, TMEM117, CAMKK2, RLN2, TIMMDC1, KLHL31*

**Table 2.** Enriched KEGG pathways identified via pathfindR analysis

ID	Pathway	p-value	Target genes
hsa04120	Ubiquitin-mediated proteolysis	<0.001	*UBA7, UBE2N, UBE2K, WWP1, TRAF6*
hsa03040	Spliceosome	<0.001	*U2SURP, SRSF2, SRSF6*
hsa04330	Notch signaling pathway	<0.001	*KAT2B, ATXN1L*
hsa05205	Proteoglycans in cancer	0.001160427	*ERBB3, CTNNB1, FZD10, EZR, WNT5A, PRKACB*
hsa04390	Hippo signaling pathway	0.001309875	*TGFBR1, GDF6, WNT5A, FZD10, YWHAE, YWHAH, CTNNB1, APC, CTNNA3*
hsa05160	Hepatitis C	0.001416289	*TRAF6, YWHAE, YWHAH, CTNNB1*
hsa04657	IL-17 signaling pathway	0.002692059	*TRAF6, ELAVL1*
hsa04144	Endocytosis	0.003025769	*AP2M1, TGFBR1, TRAF6, WWP1, PDCD6IP*
hsa03015	mRNA surveillance pathway	0.003942335	*NUDT21, CPSF6, CPSF7, UPF2*
hsa05225	Hepatocellular carcinoma	0.008091156	*TGFA, TGFBR1, WNT5A, FZD10, APC, CTNNB1, SMARCE1, ARID2*
hsa04670	Leukocyte transendothelial migration	0.008953354	*EZR, CTNNB1, CTNNA3*
hsa03013	RNA transport	0.0091471	*NUP50, POM121, EIF1AY, EIF1AX, EIF4E2, EIF4EBP2, UPF2*
hsa05203	Viral carcinogenesis	0.011431025	*CREB5, YWHAE, YWHAH, REL, PRKACB, GTF2H3, CDKN1B, KAT2B, IL6ST*
hsa04919	Thyroid hormone signaling pathway	0.014664218	*PRKACB, KAT2B, CTNNB1*
hsa04520	Adherens junction	0.015121939	*CTNNB1, CTNNA3, YES1, TGFBR1*
hsa05226	Gastric cancer	0.016356287	*WNT5A, FZD10, APC, CTNNB1, CDKN1B, TGFBR1, CTNNA3*
hsa05230	Central carbon metabolism in cancer	0.017277363	*PDK1, RET*
hsa04550	Signaling pathways regulating pluripotency of stem cells	0.017619136	*IL6ST, WNT5A, FZD10, APC, CTNNB1*
hsa04621	NOD-like receptor signaling pathway	0.018069616	*TRAF6, NLRP1, YWHAE*
hsa04934	Cushing syndrome	0.030370611	*PRKACB, CREB5, CDKN1B, WNT5A, FZD10, CTNNB1, APC*
hsa04310	Wnt signaling pathway	0.033429078	*WNT5A, FZD10, CTNNB1, APC, PRKACB, PPP3R1*
hsa05131	Shigellosis	0.034316691	*TRAF6, UBE2N*
hsa05216	Thyroid cancer	0.040790494	*RET, CTNNB1*
hsa05167	Kaposi sarcoma-associated herpesvirus infection	0.045496474	*GNB4, PPP3R1, IL6ST, CTNNB1*

ID indicates the Kyoto Encyclopedia of Genes and Genomes (KEGG) pathway ID. The pathway column indicates the name of pathways. The p-value column indicates the lowest enrichment p-value obtained through multiple iterations. The target genes column indicates the target genes (that are the targets of at least one miRNA) involved in the given enriched pathway.

**Table 3.** Overall survival and pathological stage analysis of the differentially expressed target genes regulated by circulating validated miRNAs in TCGA and GTEx datasets

Circulating validated miRNAs	Differentially expressed target genes	miRDB score	Differential expression analysis		Overall survival analysis		Pathological stage analysis	
			Log2 (fold change)	adjp	Log-rank p-value	FDR	Pr(>F)	FDR
Hsa-miR-885-5p	*GALNT3*	100	2.444	3.13E-53	0.41	0.99	0.565	0.649142857142857
	*LRP4*	95	‒1.867	2.18E-17	0.86	0.99	0.86	0.917333333333333
	*SYTL4*	95	‒4.934	6.99E-181	0.96	0.99	0.54	0.649142857142857
	*LCP1*	93	2.188	7.68E-31	0.83	0.99	0.0715	0.165257142857143
	*C8orf88*	92	‒2.239	6.46E-65	0.059	0.618666666666667	0.88	0.92327868852459
	*ADD1*	92	‒1.492	3.55E-50	0.85	0.99	0.313	0.435478260869565
	*MAN1C1*	91	‒2.329	6.38E-61	0.053	0.618666666666667	0.67	0.749241379310345
	*ECE1*	90	‒1.596	1.17E-32	0.0067	0.4288	0.0063	0.0310153846153846
	*MAP4K3*	89	‒1.158	3.69E-35	0.98	0.99	0.298	0.434909090909091
	*ATAD2B*	89	‒1.679	6.85E-71	0.37	0.99	0.0048	0.02832
	*KAT2B*	88	‒1.820	8.32E-63	0.087	0.618666666666667	0.0365	0.106181818181818
	*EMILIN2*	88	‒1.063	5.2E-17	0.88	0.99	0.568	0.649142857142857
	*NLRP1*	88	‒3.098	2.14E-77	0.91	0.99	0.297	0.434909090909091
	*CEP170*	87	‒1.235	1.02E-34	0.97	0.99	0.0222	0.07104
	*PDE10A*	86	‒2.166	6.35E-91	0.51	0.99	0.716	0.776677966101695
	*ZC3H12C*	86	‒1.207	5.94E-52	0.31	0.99	0.129	0.264
	*PCSK5*	86	‒2.428	1.61E-73	0.76	0.99	0.307	0.435478260869565
	*LAMC2*	84	3.724	4.23E-83	0.98	0.99	0.461	0.59008
	*MACF1*	84	‒1.498	2.12E-35	0.072	0.618666666666667	0.0103	0.0406588235294118
	*ZNF407*	84	‒1.085	2.9E-54	0.033	0.618666666666667	0.00033	0.017856
	*PHF6*	84	‒1.270	2.27E-32	0.69	0.99	0.0477	0.123648
	*SRSF6*	83	‒1.279	3.03E-43	0.83	0.99	0.0483	0.123648
	*TPMT*	83	1.519	9.14E-51	0.33	0.99	0.00528	0.02832
	*CEBPD*	83	‒1.953	6.27E-35	0.051	0.618666666666667	0.266	0.4256
	*ZNF10*	82	2.257	9.93E-84	0.8	0.99	0.00231	0.02112
	*SEMA6A*	82	‒2.468	3.3E-52	0.27	0.99	0.107	0.228266666666667
	*UBE2N*	82	1.075	2.46E-25	0.92	0.99	0.0881	0.194427586206897
	*CXADR*	82	3.495	4.19E-107	0.23	0.99	0.132	0.264
	*CDK17*	81	‒1.058	2.1E-28	0.63	0.99	0.0108	0.0406588235294118
	*TMEM185B*	81	1.771	1.01E-63	0.46	0.99	0.158	0.297411764705882
	*ZNF516*	81	‒2.404	6.02E-72	0.99	0.99	0.00876	0.0400457142857143
	*HMBOX1*	80	‒1.798	1.66E-54	0.9	0.99	0.249	0.408615384615385
	*SAMD12*	80	1.632	1.01E-56	0.77	0.99	0.521	0.649142857142857
	*CLASP2*	79	‒1.691	1.63E-52	0.6	0.99	0.00269	0.02152
	*LRRK2*	79	2.551	1.75E-156	0.72	0.99	0.243	0.408615384615385
	*LYPLA1*	79	1.886	6.21E-55	0.89	0.99	0.0723	0.165257142857143
	*HSBP1*	78	1.049	7.63E-27	0.031	0.618666666666667	0.942	0.963
	*TTC30A*	77	1.336	1.37E-39	0.61	0.99	0.00212	0.02112
	*ANKFY1*	77	‒1.215	3.2E-28	0.47	0.99	0.000853	0.0181973333333333
	*UBE2K*	77	1.396	3.47E-32	0.3	0.99	0.18	0.311351351351351
	*NR2C1*	77	‒1.060	8.37E-32	0.6	0.99	0.0633	0.155815384615385
	*GOPC*	76	‒1.215	5.91E-36	0.45	0.99	0.431	0.562938775510204
	*HRASLS5*	76	‒1.137	2.09E-46	0.96	0.99	0.963	0.963
	*SLC38A2*	76	‒1.343	3.99E-26	0.98	0.99	0.18	0.311351351351351
	*RBPMS*	75	‒2.988	1.62E-66	0.62	0.99	0.679	0.749241379310345
	*DGKH*	75	‒1.085	6.98E-54	0.78	0.99	0.151	0.292848484848485
	*CREBZF*	74	‒2.524	3.14E-80	0.87	0.99	0.0133	0.0448
	*TRIM7*	74	‒1.351	5.49E-71	0.95	0.99	0.297	0.434909090909091
	*IL6ST*	73	‒1.746	4.03E-55	0.61	0.99	0.347	0.472510638297872
	*VPS41*	73	‒1.177	4.94E-27	0.47	0.99	0.00946	0.0403626666666667
	*JAKMIP3*	73	‒1.807	2.5E-194	0.77	0.99	0.567	0.649142857142857
	*FZD10*	71	1.693	1.78E-14	0.27	0.99	0.299	0.434909090909091
	*HGSNAT*	71	‒1.255	3.27E-27	0.66	0.99	0.00128	0.02048
	*TNRC6C*	71	‒1.140	1.25E-20	0.2	0.99	0.533	0.649142857142857
	*SEMA6D*	70	‒1.887	3.05E-42	0.6	0.99	0.382	0.509333333333333
	*MYSM1*	69	‒1.920	1.94E-75	0.66	0.99	0.0123	0.0437333333333333
	*TBCC*	69	1.468	7.57E-48	0.08	0.618666666666667	0.169	0.309028571428571
	*CENPK*	69	1.782	2.7E-57	0.7	0.99	0.00474	0.02832
	*PLEKHA5*	68	‒1.640	9.96E-46	0.99	0.99	0.0351	0.106181818181818
	*ABHD17C*	68	2.353	7E-69	0.97	0.99	0.0425	0.118260869565217
	*UBN2*	68	‒1.158	1.26E-39	0.57	0.99	0.000558	0.017856
	*FAM126A*	67	‒1.282	5.07E-34	0.55	0.99	0.948	0.963
	*MIER3*	67	‒1.139	1.5E-37	0.79	0.99	0.00531	0.02832
	*MDM4*	67	‒1.924	5.04E-60	0.99	0.99	0.00181	0.02112
Hsa-miR-1909-5p	*RCBTB2*	95	‒1.508	2.75E-43	0.33	0.727272727272727	0.645	0.903333333333333
	*MEF2C*	95	‒2.096	3.29E-71	0.75	0.833333333333333	0.974	0.978
	*BCO2*	88	‒1.333	5.48E-43	0.4	0.727272727272727	0.807	0.903333333333333
	*C4orf19*	83	1.547	5.38E-31	0.39	0.727272727272727	0.433	0.721666666666667
	*ZNF275*	81	‒1.501	1.58E-30	0.87	0.89	0.022	0.0733333333333333
	*PIGZ*	80	1.131	2.34E-20	0.63	0.833333333333333	0.415	0.721666666666667
	*ERBB3*	80	4.024	2.49E-87	0.13	0.727272727272727	0.793	0.903333333333333
	*METTL7B*	78	3.511	3.47E-77	0.19	0.727272727272727	0.609	0.903333333333333
	*KCNK15*	77	3.713	7.25E-70	0.27	0.727272727272727	0.978	0.978
	*GGA2*	76	‒1.710	1.26E-43	0.89	0.89	0.0143	0.0628
	*CEMP1*	75	‒1.950	6.66E-67	0.32	0.727272727272727	0.417	0.721666666666667
	*GTPBP1*	74	1.18	1.68E-34	0.44	0.733333333333333	0.00195	0.0195
	*TRIO*	74	‒1.925	2.19E-54	0.71	0.833333333333333	0.0012	0.0195
	*MEOX2*	73	‒1.228	3.36E-33	0.025	0.5	0.0157	0.0628
	*DICER1*	72	‒1.709	6.59E-51	0.68	0.833333333333333	0.0073	0.0486666666666667
	*SGCD*	72	‒1.537	6.45E-35	0.67	0.833333333333333	0.715	0.903333333333333
	*TGFA*	71	1.493	1.38E-30	0.11	0.727272727272727	0.813	0.903333333333333
	*UBA7*	70	‒1.552	1.11E-30	0.17	0.727272727272727	0.0311	0.0888571428571429
	*ECHDC2*	70	‒3.671	4.08E-118	0.75	0.833333333333333	0.123	0.273333333333333
	*CDKN1B*	69	‒1.130	1.33E-30	0.3	0.727272727272727	0.0933	0.23325
Hsa-let-7d-3p	*PTGIS*	88	‒2.804	5.93E-41	0.18	0.54	0.199	0.3582
	*HMGA2*	86	1.875	6.14E-18	0.073	0.3285	0.243	0.3645
	*GSTK1*	81	1.034	1.13E-26	0.25	0.5625	0.0408	0.1224
	*KANSL1*	74	‒1.149	5.13E-28	0.47	0.74	0.00972	0.04374
	*NOM1*	72	‒1.178	2.34E-31	0.73	0.74	0.00311	0.02799
	*ABCA8*	72	‒5.852	1.13E-253	0.74	0.74	0.556	0.714857142857143
	*CASD1*	71	‒1.808	4.08E-81	0.65	0.74	0.133	0.29925
	*RLN2*	69	1.1	1.26E-26	0.054	0.3285	0.74	0.8325
	*TIMMDC1*	67	1.527	1.44E-60	0.65	0.74	0.897	0.897

TCGA, The Cancer Genome Atlas; GTEx, Genotype-Tissue Expression; FDR, false discovery rate.
